# Cord pilot trial, comparing alternative policies for timing of cord clamping before 32 weeks gestation: follow-up for women up to one year

**DOI:** 10.1186/s12884-019-2223-9

**Published:** 2019-02-21

**Authors:** Lucy Bradshaw, Alexandra Sawyer, Lindsay Armstrong-Buisseret, Eleanor Mitchell, Susan Ayers, Lelia Duley

**Affiliations:** 1 Nottingham Clinical Trials Unit, University of Nottingham, Nottingham, NG7 2UH UK; 2 School of Health Sciences, University of Brighton, Falmer, BN1 9PH UK; 3 Centre for Maternal and Child Health, School of Health Sciences, City University London, London, EC1V 0HB UK

**Keywords:** Preterm birth, Umbilical cord clamping, Neonatal care with cord intact, Clinical trial, Follow up for women

## Abstract

**Background:**

The Cord Pilot Trial compared two alternative policies for cord clamping at very preterm birth at eight UK maternity units: clamping after at least 2 min and immediate neonatal care (if needed) with cord intact, or clamping within 20 s and neonatal care after clamping. This paper reports follow-up of the women by two self-completed questionnaires up to one year after the birth.

**Methods:**

Women were given or posted the first questionnaire between four and eight weeks after birth, usually before their baby was discharged, and were posted a second similar questionnaire at one year. The questionnaire included the Hospital Anxiety and Depression Scale; the Preterm Birth Experience and Satisfaction Scale (P-BESS) and questions about their baby’s feeding.

**Results:**

Of 261 women randomised (132 clamping ≥2 min, 129 clamping ≤20 s), six were excluded as birth was after 35^+ 6^ weeks (2, 4 in each group respectively). Six were not sent either questionnaire. The first questionnaire was given/sent to 244 and returned by 186 (76%) (79, 74%). The second, at one year, was sent to 242 and returned by 133 (55%) (66, 43%).

On the first questionnaire, 89 (49%) had a score suggestive of an anxiety disorder, and 55 (30%) had a score suggestive of depression. Satisfaction with care at birth was high: median total P-BESS score 77 [interquartile range 68 to 84] (scale 17 to 85). There was no clear difference in anxiety, depression, or satisfaction with care between the two allocated groups. The median number of weeks after birth women breastfed/expressed was 16 (95% confidence interval (CI) 13 to 20, *n* = 119) for those allocated clamping ≥2 min and 12 (95% CI 11 to 16, *n* = 103) for those allocated clamping ≤20 s.

**Conclusions:**

The response rate was higher for the earlier questionnaire than at one year. A high proportion of women reported symptoms of anxiety or depression, however there were no clear differences between the allocated groups. Most women reported that they had breastfed or expressed milk and those allocated deferred cord clamping reported continuing this for slightly longer.

**Trial registration:**

ISRCTN 21456601, registered 28th February 2013, http://www.isrctn.com/ISRCTN21456601

**Electronic supplementary material:**

The online version of this article (10.1186/s12884-019-2223-9) contains supplementary material, which is available to authorized users.

## Background

Preterm birth is a major determinant of adverse infant outcome in terms of survival, quality of life, psychosocial and emotional impact on the family, and costs for health services [1–3]. Evidence from randomised trials suggests that, for infants born preterm, deferring cord clamping may improve outcome at discharge from hospital [4, 5]. To date, to our knowledge, none of these studies have reported outcomes for women after discharge from hospital after the birth.

The Cord Pilot Trial was a randomised comparison of alternative policies for management of cord clamping at very preterm birth [6–8]. Outcomes to hospital discharge for the women and their babies have been reported [8]. Traditionally, at birth very preterm infants are taken to the resuscitation equipment; which is usually at the side of the room or in another room. To allow deferred cord clamping without delaying neonatal care for babies requiring resuscitation at birth, newborn life support at birth was provided with cord intact; this also allowed women to share the first moments of their baby’s life. Providing neonatal care at birth beside the mother is valued by parents [9] and acceptable to clinicians [10]. For the women, our hypothesis was that deferred cord clamping and providing immediate neonatal care (if needed) with cord intact beside the mother might facilitate bonding, potentially improving emotional outcomes, breastfeeding and satisfaction with care. This paper reports follow-up by self-completed questionnaire and outcomes for the women up to one year after the birth.

## Methods

The Cord Pilot Trial compared cord clamping after at least two minutes and providing immediate neonatal care, if needed, with cord intact (deferred clamping), with clamping within 20 s and neonatal care after clamping (immediate clamping) at very preterm birth (before 32 weeks gestation). The trial was conducted at eight UK tertiary maternity units. The protocol is published [6, 7]. Initially, the objective was to assess the feasibility of conducting a large multicentre UK trial. When feasibility was demonstrated, recruitment continued whilst funding for the full trial was sought. Recruitment closed when the funding application for the full trial was unsuccessful [11].

Two different consent pathways were used within the trial [6, 12]. Where possible the usual written consent process was used (one stage consent pathway). However if birth was imminent and there was insufficient time for the usual consent process, women could give oral assent to the trial prior to birth. After the birth, written consent for participation in follow up was sought from the women who provided oral assent (two stage consent pathway).

Women were asked to complete two similar questionnaires, the first between four and eight weeks after giving birth, the second at one year. We initially planned the first questionnaire would be posted to the woman’s home six weeks after the birth, but this often coincided with discharge of the baby which was not a good time for the women to receive it. Therefore, we changed this and if the baby was still in hospital at age four weeks, the research midwife/nurse gave the questionnaire to the woman when she was visiting. If the baby was discharged by age four weeks, we posted it to her when the baby was eight weeks old (or if the baby died, eight weeks after the birth). We posted a similar questionnaire to women at one year, along with a birthday card for the child (not sent if the child died). A stamped addressed envelope was provided to return completed questionnaires. If there was no response, we sent a postal reminder after two weeks. If there was still no response after another two weeks, we telephoned the woman and offered the opportunity to complete the questionnaire over the telephone. If no telephone number was available, we sent a second postal reminder. Before attempting to contact women, the study team checked contact details with the recruiting site. If contact could not be made, a letter was sent to the GP to check if the woman’s contact details had changed. If their baby had died and the woman did not respond, she was not contacted with reminders.

### Questions used

The questionnaire included the Hospital Anxiety and Depression Scale (HADS) [13] to assess depression and anxiety; the Preterm Birth Experience and Satisfaction Scale (P-BESS) [14] to assess satisfaction with care at birth; and a section asking women about their baby’s feeding (not included if the baby had died). A final section asked about women’s experiences of participating in the trial, and the responses to these questions are reported separately [submitted for publication].

The HADS includes 14 questions, seven about anxiety and seven about depression in the past week, and has been used previously for parents of preterm babies [15]. Each question is scored from zero to three, and the seven items in each subscale are summed to create a score ranging from zero to 21 for either anxiety or depression. Higher scores indicate increased symptoms of anxiety or depression; a score of eight or more being suggestive of a mood disorder [16]. If a participant missed one item in the subscale, we used the mean of the completed items in that subscale to impute the missing item to calculate the subscale score. If more than one item was missing, the subscale score was not calculated. The P-BESS was developed specifically to assess parent’s satisfaction with care during very preterm birth and has 17 items each with a five point response scale (strongly disagree to strongly agree coded 1 to 5, some items reversed), in three subscales: interpersonal care (7 items, range 7 to 35), information and explanations (7 items, range 7 to 35), and confidence in staff (3 items, range 3 to 15). Missing items in each subscale were imputed using the mean of the completed items if 1 or 2 items were missing for the interpersonal care or information and explanations subscale or 1 item was missing for the confidence in staff subscale. The total score ranges between 17 and 85, with higher scores indicating greater satisfaction. We also asked whether the woman had ever breastfed or expressed milk for their baby and, if so, whether they were continuing to do so or the age of the baby when breastfeeding/expressing stopped.

#### Outcomes

For the initial feasibility trial, outcomes were the proportion of women responding and the reasons for losses to follow-up. Clinical outcomes were depression (HADS score ≥ 8); anxiety (HADS score ≥ 8); P-BESS total score and subscale scores; and for women who were breast feeding/expressing at discharge, duration of breast feeding/expressing.

#### Analysis

All analyses were based on the groups as randomly allocated (intention to treat). Women who gave birth after 35^+ 6^ weeks gestation were excluded from the analysis of outcomes to discharge, as outcome for these babies are different from those born very preterm [8]. They are therefore also excluded here. Response rates for the two questionnaires were described, along with reasons for losses to follow-up. Baseline and characteristics at birth for women who did not respond were explored. Scores on the HADS and P-BESS questionnaires were summarised descriptively. Duration of breast feeding/expressing from birth was described using Kaplan Meier curves for women who had ever breastfed/expressed and their baby survived to one year, censoring at the last known time of breastfeeding/expressing.

## Results

**Fig. 1:**
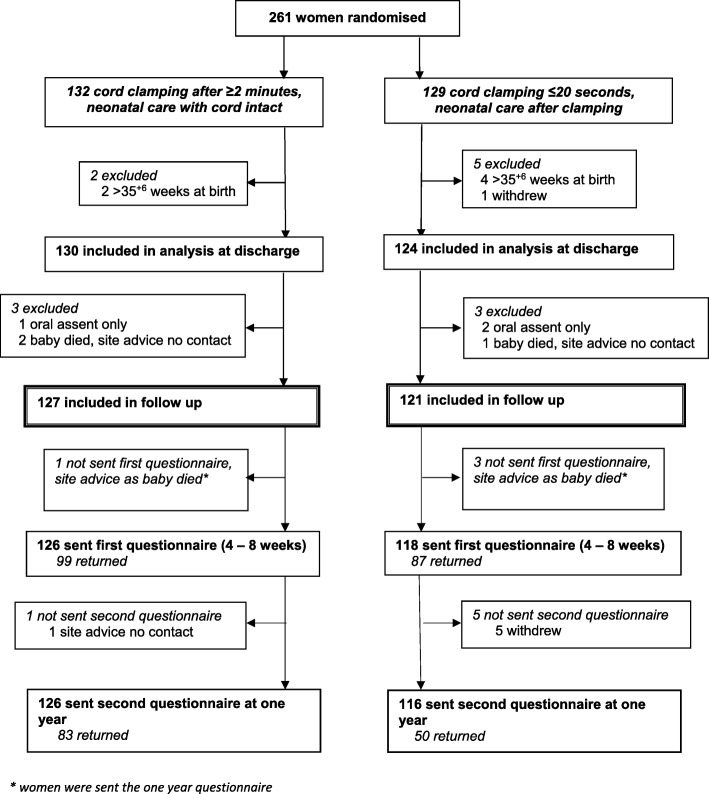
CONSORT flow for the follow up of women to one year with questionnaires

Overall 261 women were randomised between March 2013 and February 2015, six of whom were excluded from the analysis as they gave birth after 35^+ 6^ weeks and one withdrew use of data, leaving 254 women included in analysis at discharge (Fig. 1). Six women were not sent either questionnaire; three of whom gave oral assent only, so consent for further follow-up was not available [8], and three whose baby died before discharge and the site advised no further contact (Fig. 1). In addition, four women were not sent the first questionnaire (at four to eight weeks) but were sent the second at one year, as the site advised us to defer follow-up to one year as their baby had died. Of the 244 women sent/given the first questionnaire, 149 (61%) were handed this before their baby was discharged home. Five women sent the first questionnaire requested no further contact, one of whom returned this questionnaire completed and four who did not. A sixth woman was not sent the one year questionnaire as the site advised us not to contact her due to safeguarding issues.

Overall, of the first questionnaires 186 of 244 (76%) were returned, along with 133 of 242 (55%) of the second at one year (Fig. 1). Women allocated deferred clamping returned a higher proportion of the first questionnaires than those allocated immediate clamping (79% versus 74% respectively), and this difference was more marked for the second questionnaire at one year (66% versus 43%). Two women sent the one year questionnaire requested no further contact from the trial. All sections were completed for 95% (176/186) of the first questionnaires returned, and 98% (130/133) of those at one year. Both questionnaires were completed by 122 women; 64 returned the first only and 11 returned the second only. Response was higher for women aged 30 or older at the time they gave consent, and for those recruited during their first pregnancy lasting 20 weeks or more (Additional file 1). Response was similar according to consent pathway (one stage or two stage), pregnancy type (singleton/twin) and gestation at birth (Additional file 1).

Baseline characteristics, birth, and outcome at discharge for women who responded were mostly similar in the two groups (Table 1). However a smaller proportion of women were primiparous and a greater proportion had a caesarean section in the deferred clamping group compared to the immediate clamping group for women who returned questionnaires at each of the two time points.

**Table 1:** Characteristics at baseline and at birth for women who returned the questionnaires

	*First (at 4–8 weeks)*		*Second (at one year)*	
	Clamp ≥2 min + neonatal care with cord intact	Clamp ≤20 s + neonatal care after clamping	Clamp ≥2 min + neonatal care with cord intact	Clamp ≤20 s + neonatal care after clamping
	*n* = 99 (%)	*n* = 87 (%)	*n* = 83 (%)	*n* = 50 (%)
Age (years) mean [sd]	30.6 [5.7]	29.3 [6.6]	31.0 [5.6]	31.3 [6.3]
Gestation at birth (weeks)				
< 26	15 (15%)	8 (9%)	12 (14%)	4 (8%)
26^+ 0^–27^+ 6^	22 (22%)	16 (18%)	16 (19%)	8 (16%)
28^+ 0^–29^+ 6^	27 (27%)	34 (39%)	22 (27%)	20 (40%)
30^+ 0^ - 31^+ 6^	34 (34%)	28 (32%)	31 (37%)	16 (32%)
≥ 32	1 (1%)	1 (1%)	2 (2%)	2 (4%)
Consent pathway^a^: two stage	26 (26%)	19 (22%)	23 (28%)	11 (22%)
one stage	73 (74%)	68 (78%)	60 (72%)	39 (78%)
Primiparous	56 (57%)	61 (70%)	49 (59%)	36 (72%)
Twin pregnancy	5 (5%)^b^	8 (9%)	5 (6%)^b^	6 (12%)
Caesarean section	66 (67%)	46 (53%)	54 (65%)	28 (56%)
*For liveborn babies* ^*c*^	*(n = 102)*	*(n = 93)*	*(n = 86)*	*(n = 54)*
Died before discharge	2	2	2	0
Intraventricular haemorrhage (grade 1–4)	31 (30%)	33 (35%)	22 (26%)	20 (37%)
Severe intraventricular haemorrhage (grade 3–4)	2 (2%)	5 (5%)	1 (1%)	2 (4%)
Length of hospital stay (days) median [25th, 75th centile]	59 [41.5, 89]	61 [40, 81]	59 [38.5, 79]	58.5 [38, 83]
Receiving mother’s breast milk at discharge	59 (58%)	54 (58%)	52 (60%)	36 (67%)

Data are n (%), unless otherwise stated. sd = standard deviation

^a^For the one stage consent pathway written consent was given before randomisation. The two stage pathway was used for women when birth was imminent, with oral assent given before randomisation and written consent for follow-up after the birth

^b^For two, one twin known intrauterine death before randomisation

^c^For liveborn babies, two women in the clamp cord within 20 s group who returned both questionnaires had stillbirths

### Depression and anxiety

There was no evidence of a clear difference between the two allocated groups in either the HADS anxiety or depression scores at either time point (Table 2). For both questionnaires, scores for depression were lower than those for anxiety (Table 2). On the first questionnaire, 89 women (49%) had a score suggestive of an anxiety disorder (≥8), and 55 (30%) had a score suggestive of depression. On the second questionnaire, scores for both the anxiety and depression scales were on average one point lower than on the first questionnaire.

**Table 2:** HADS scores for anxiety and depression and P-BESS for women’s satisfaction with care at birth

	*First (at 4–8 weeks)*		*Second (at one year)*	
	Clamp ≥2 min + neonatal care with cord intact	Clamp ≤20 s + neonatal care after clamping	Clamp ≥2 min + neonatal care with cord intact	Clamp ≤20 s + neonatal care after clamping
	*n* = 99 (%)	*n* = 87 (%)	*n* = 83 (%)	*n* = 50 (%)
*HADS scores* ^*a*^				
depression mean [sd]	5.1 [3.3]	6.0 [3.7]	4.3 [3.5]	4.8 [3.0]
≥ 8	28 (29%)	27 (31%)	17 (20%)	10 (20%)
anxiety mean [sd]	7.4 [4.1]	7.6 [4.7]	6.7 [3.9]	6.2 [3.9]
≥ 8	49 (50%)	40 (47%)	34 (41%)	18 (36%)
*P-BESS scores, median [25th, 75th centile]*				
total	80 [69, 85]	76 [68, 82]	78 [67, 84]	78 [70, 82]
interpersonal care^b^	34 [29, 35]	32 [28, 34]	33 [28, 35]	33 [30, 35]
information and explanations	33 [28, 35]	31 [28, 34]	33 [27, 35]	31 [28, 34]
confidence in staff	15 [12, 15]	13 [11, 15]	14 [11, 15]	14 [12, 15]

^a^Anxiety and depression scores were reasonably normally distributed. HADS questionnaire not completed by one participant in each group on the first questionnaire and by one participant in the clamp cord ≤20 s group on the second questionnaire at one year

^b^P-BESS interpersonal scale score not fully completed by one participant in each group on the first questionnaire

### Satisfaction with care at birth

Satisfaction with care at birth as measured by the P-BESS was high (Table 2). There was no evidence of a clear difference between the two groups, and scores were similar at the two timepoints. Scores were left skewed, with a small proportion of women in both groups reporting much lower satisfaction. In the free text responses, aspects of care at birth that parents were particularly satisfied with were: staff calmness, staff explaining what was happening (including when the situation changed), and being given the opportunity to see their baby. For women reporting much lower satisfaction scores, free text responses included staff not listening to what they said about their labour.

### Breast-feeding

**Fig. 2:**
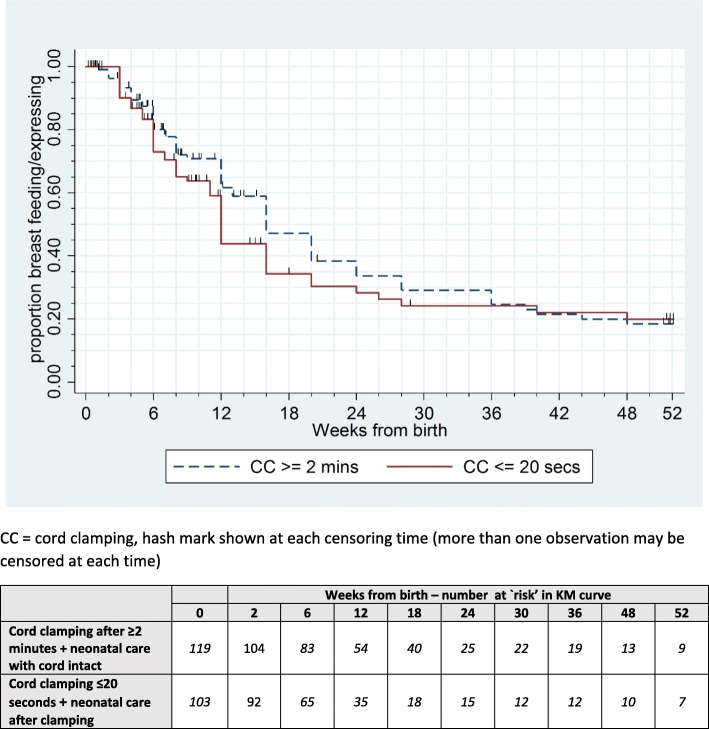
Kaplan-Meier curves to show baby age in weeks when women stopped breastfeeding/expressing

Of women with a baby alive at one year, almost all reported ever breastfeeding or expressing breast milk (Table 3). The duration of breastfeeding or expressing was slightly longer for women allocated deferred clamping (median 16 weeks after birth, 95% confidence interval (CI) 13 to 20) than for those allocated immediate clamping (median 12 weeks after birth, 95% CI 11 to 16) (Table 3 and Fig. 2).

**Table 3:** Breast feeding/expressing breast milk for women with a baby alive at one year

	Clamp ≥2 min + neonatal care with cord intact (*n* = 122)	Clamp ≤20 s + neonatal care after clamping (*n* = 112)
ever breast fed/expressed	119 (98%)	104 (93%)
median weeks from birth to stopping breastfeeding/expressing^a^, Kaplan Meier survival estimates [95% CI]	16 [13 to 20]	12 [11 to 16]

^a^Clamp ≥2 min + neonatal care with cord intact (*n* = 119) Clamp ≤20 s + neonatal care after clamping (*n* = 103)

## Discussion

The Cord Pilot Trial used two self-completed questionnaires, at four to eight weeks and at one year, to ask women about symptoms of anxiety and depression, their satisfaction with care at birth, and whether they had breast fed or expressed milk. Overall, three quarters of women responded to the first questionnaire, with similar proportions responding in the two allocated groups. Response to the second questionnaire at one year was lower, with just over half the women returning a completed questionnaire. At one year, a much higher percentage of women allocated deferred clamping responded (66%) than those allocated immediate clamping (43%), possibly due to women in the immediate clamping group feeling they were not part of the trial as they received usual care [12]. This low response and differential follow-up between the two groups limits comparisons of outcomes.

The prevalence of possible anxiety and depression was high, with symptoms of anxiety more prevalent than symptoms of depression. This is unsurprising, as anxiety and depression are common problems after childbirth [17] particularly in parents of babies who are born very preterm [18]. Estimates of the magnitude of the difference between mothers of term and preterm births vary [19]. Anxiety and depression scores in this trial were similar in the two allocated groups however. This suggests that these symptoms are not influenced by the policy for cord management at birth.

Most women in both groups reported high satisfaction with their care at the birth. This is consistent with high satisfaction with care at birth found in a national UK survey across a range of gestational ages at birth [20] and in the P-BESS development study [14]. This is the first study to explore satisfaction with care following preterm birth in the UK using a measure specifically designed for preterm birth. Responses to the open ended questions highlight the value of using a measure appropriate for preterm birth; as women frequently mentioned staff calmness as an aspect of care they were particularly satisfied with, and this is not included in measures of satisfaction at term birth [21].

Support for women to express milk and breast feed is usual care at UK neonatal units, and so was available in the trial to women in both groups. Most women reported that they had breast fed or expressed breast milk for their baby. This was slightly higher for those allocated deferred clamping, and these women also continued for longer than those allocated immediate cord clamping. This supports our hypothesis that deferring clamping and providing immediate neonatal care, if needed, with cord intact might facilitate bonding at birth as women report that they value being able to see and touch their baby at very preterm birth [9]. Nevertheless, confirmation in a larger study is required.

The first few weeks after returning home with their new baby can be a stressful time for parents, particularly if the baby was premature. Clearly this is not a good time for completing a postal questionnaire. We quickly adjusted our study so that women were either given the questionnaires whilst visiting their baby in the neonatal unit, if the baby was still there at four weeks, or were sent it by post at eight weeks. Response to this first questionnaire was high. Response to the second was lower. Various factors are likely to have contributed to this: once women agreed to participate, the trial was relatively minor event compared to birth of their preterm baby [12] and so the memory of it will have faded; women may not have known or remembered how long they would be staying in the trial or that that they would be asked to complete questionnaires [12]; and they may not have realised that the trial aimed to follow them up as well as their babies. Strategies to evaluate in future similar trials to improve follow up are reminding women and giving them more information about what will happen for follow up after they have been recruited, and explaining how their data will be used. These could include discussion before they are discharged, newsletters, websites and social media.

The low response for women allocated immediate clamping to the second questionnaire at one year may have been due to factors such as disappointment with their allocated intervention due to a preference for deferred clamping, or a misunderstanding of randomisation as some women allocated immediate clamping felt they would not contribute to the trial, or had somehow ‘failed’ the study [12]. Difficulty in understanding randomisation has been widely identified as a problem for consent in randomised trials [22, 23]. Strategies to explain randomisation in meaningful ways to potential trial participants should be developed and evaluated, along with improved training on how to offer participation and consent. Although the differential response does not appear to be related to characteristics at baseline, it nevertheless introduces potential for bias. This underlines the importance of minimising losses to follow-up so that potential for bias in assessment of outcome is minimised.

## Conclusions

Response to the first questionnaire at four to eight weeks was higher than for the second at one year. Satisfaction with care at birth was high, as were scores suggestive of an anxiety disorder and/or a depressive disorder. Most women reported that they had breastfed or expressed milk for their baby, and those allocated deferred cord clamping reported continuing this for slightly longer.

## Additional file

Additional file 1: Comparison of baseline characteristics according to questionnaire completion. (DOCX 36 kb)

## Abbreviations

CI: Confidence interval
HADS: Hospital Anxiety and Depression Scale
P-BESS: Preterm Birth Experience and Satisfaction Scale
SD: Standard deviation
