# Supplementary material for: Cord pilot trial, comparing alternative policies for timing of cord clamping before 32 weeks gestation: follow-up for women up to one year
Source: BMC Pregnancy Childbirth. 2019 Feb 21;19:78. doi: 10.1186/s12884-019-2223-9 (PMC6383279; doi:10.1186/s12884-019-2223-9)
Supplement: Supplementary file 1 — Comparison of baseline characteristics according to questionnaire completion. (DOCX 36 kb) [file 12884_2019_2223_MOESM1_ESM.docx]

**Additional file 1: Comparison of baseline characteristics according to questionnaire completion.**

Table S1 Baseline characteristics according to completion of the first questionnaire (4 – 8 weeks) and allocated group

*For each level of the characteristic, the table shows the % not completing/completing the first questionnaire in each group and overall (i.e. row %)*

| **Characteristic** | **Clamp ≥2 minutes + neonatal care with cord intact**  **n = 130** | | **Clamp ≤20 seconds + neonatal care after clamping**  **n = 124** | | **Both groups**  **(n = 254)** | |
| --- | --- | --- | --- | --- | --- | --- |
|  | **Not sent/not completed**  **n = 31** | **Completed**  **n = 99** | **Not sent/not completed**  **n = 37** | **Completed**  **n = 87** | **Not sent/not completed**  **n = 68** | **Completed**  **n = 186** |
| Recruitment period |  |  |  |  |  |  |
| First year (feasibility) | 13 (23%) | 44 (77%) | 18 (28%) | 47 (72%) | 31 (25%) | 91 (75%) |
| Second year | 18 (25%) | 55 (75%) | 19 (32%) | 40 (68%) | 37 (28%) | 95 (72%) |
|  |  |  |  |  |  |  |
| Age of mother at consent |  |  |  |  |  |  |
| < 20 | 3 (60%) | 2 (40%) | 4 (36%) | 7 (64%) | 7 (44%) | 9 (56%) |
| 20 to 24 | 6 (30%) | 14 (70%) | 5 (26%) | 14 (74%) | 11 (28%) | 28 (72%) |
| 25 to 29 | 8 (30%) | 19 (70%) | 9 (30%) | 21 (70%) | 17 (30%) | 40 (70%) |
| 30 to 34 | 9 (18%) | 41 (82%) | 9 (24%) | 29 (76%) | 18 (20%) | 70 (80%) |
| 35 to 39 | 2 (10%) | 18 (90%) | 10 (50%) | 10 (50%) | 12 (30%) | 28 (70%) |
| ≥ 40 | 3 (38%) | 5 (63%) | - | 6 (100%) | 3 (21%) | 11 (79%) |
|  |  |  |  |  |  |  |
| Gestation at birth |  |  |  |  |  |  |
| <26 weeks | 6 (29%) | 15 (71%) | 6 (43%) | 8 (57%) | 12 (34%) | 23 (66%) |
| 26 weeks to 27^6^ weeks | 3 (12%) | 22 (88%) | 5 (24%) | 16 (76%) | 8 (17%) | 38 (83%) |
| 28 weeks to 29^6^ weeks | 8 (23%) | 27 (77%) | 9 (21%) | 34 (79%) | 17 (22%) | 61 (78%) |
| 30 weeks to 31^6^ weeks | 13 (28%) | 34 (72%) | 16 (36%) | 28 (64%) | 29 (32%) | 62 (68%) |
| ≥ 32 weeks | 1 (50%) | 1 (50%) | 1 (50%) | 1 (50%) | 2 (50%) | 2 (50%) |
|  |  |  |  |  |  |  |
|  |  |  |  |  |  |  |
|  |  |  |  |  |  |  |
| Consent pathway |  |  |  |  |  |  |
| Usual one stage | 24 (25%) | 73 (75%) | 20 (23%) | 68 (77%) | 44 (24%) | 141 (76%) |
| Two stage | 7 (21%) | 26 (79%) | 17 (47%) | 19 (53%) | 24 (35%) | 45 (65%) |
|  |  |  |  |  |  |  |
| Pregnancy type |  |  |  |  |  |  |
| Singleton | 29 (24%) | 94 (76%) | 35 (31%) | 79 (69%) | 64 (27%) | 173 (73%) |
| Twin | 2 (29%) | 5 (71%) | 2 (20%) | 8 (80%) | 4 (24%) | 13 (76%) |
|  |  |  |  |  |  |  |
| First pregnancy lasting 20 weeks or more |  |  |  |  |  |  |
| No | 18 (30%) | 43 (70%) | 23 (47%) | 26 (53%) | 41 (37%) | 69 (63%) |
| Yes | 13 (19%) | 56 (81%) | 14 (19%) | 61 (81%) | 27 (19%) | 117 (81%) |
|  |  |  |  |  |  |  |
| Mode of delivery |  |  |  |  |  |  |
| Caesarean | 16 (20%) | 66 (80%) | 21 (31%) | 46 (69%) | 37 (25%) | 112 (75%) |
| Vaginal | 15 (31%) | 33 (69%) | 16 (28%) | 41 (72%) | 31 (30%) | 74 (70%) |
|  |  |  |  |  |  |  |
| Breast feeding/expressing at their discharge |  |  |  |  |  |  |
| No | 2 (40%) | 3 (60%) | 4 (44%) | 5 (56%) | 6 (43%) | 8 (57%) |
| Yes | 25 (21%) | 96 (79%) | 30 (27%) | 81 (73%) | 55 (24%) | 177 (76%) |
| Baby not alive at mother’s discharge | 4 (100%) | - | 3 (75%) | 1 (25%) | 7 (88%) | 1 (13%) |

Table S2 Baseline characteristics according to completion of the second questionnaire (one year) and allocated group

*For each level of the characteristic, the table shows the % not completing/completing the second questionnaire in each group and overall (i.e. row %)*

| **Characteristic** | **Clamp ≥2 minutes + neonatal care with cord intact**  **n = 130** | | **Clamp ≤20 seconds + neonatal care after clamping**  **n = 124** | | **Both groups**  **(n = 254)** | |
| --- | --- | --- | --- | --- | --- | --- |
|  | **Not sent/not completed**  **n = 47** | **Completed**  **n = 83** | **Not sent/not completed**  **n = 74** | **Completed**  **n = 50** | **Not sent/not completed**  **n = 121** | **Completed**  **n = 133** |
| Recruitment period |  |  |  |  |  |  |
| First year (feasibility) | 18 (32%) | 39 (68%) | 33 (51%) | 32 (49%) | 51 (42%) | 71 (58%) |
| Second year | 29 (40%) | 44 (60%) | 41 (69%) | 18 (31%) | 70 (53%) | 62 (47%) |
|  |  |  |  |  |  |  |
| Age of mother at consent |  |  |  |  |  |  |
| < 20 | 4 (80%) | 1 (20%) | 10 (91%) | 1 (9%) | 14 (88%) | 2 (13%) |
| 20 to 24 | 9 (45%) | 11 (55%) | 13 (68%) | 6 (32%) | 22 (56%) | 17 (44%) |
| 25 to 29 | 14 (52%) | 13 (48%) | 18 (60%) | 12 (40%) | 32 (56%) | 25 (44%) |
| 30 to 34 | 12 (24%) | 38 (76%) | 22 (58%) | 16 (42%) | 34 (39%) | 54 (61%) |
| 35 to 39 | 5 (25%) | 15 (75%) | 11 (55%) | 9 (45%) | 16 (40%) | 24 (60%) |
| ≥ 40 | 3 (38%) | 5 (63%) | - | 6 (100%) | 3 (21%) | 11 (79%) |
|  |  |  |  |  |  |  |
| Gestation at birth |  |  |  |  |  |  |
| <26 weeks | 9 (43%) | 12 (57%) | 10 (71%) | 4 (29%) | 19 (54%) | 16 (46%) |
| 26 weeks to 27^6^ weeks | 9 (36%) | 16 (64%) | 13 (62%) | 8 (38%) | 22 (48%) | 24 (52%) |
| 28 weeks to 29^6^ weeks | 13 (37%) | 22 (63%) | 23 (53%) | 20 (47%) | 36 (46%) | 42 (54%) |
| 30 weeks to 31^6^ weeks | 16 (34%) | 31 (66%) | 28 (64%) | 16 (36%) | 44 (48%) | 47 (52%) |
| ≥ 32 weeks | - | 2 (100%) | - | 2 (100%) | - | 4 (100%) |
|  |  |  |  |  |  |  |
|  |  |  |  |  |  |  |
|  |  |  |  |  |  |  |
| Consent type |  |  |  |  |  |  |
| Usual one stage | 37 (38%) | 60 (62%) | 49 (56%) | 39 (44%) | 86 (46%) | 99 (54%) |
| Two stage | 10 (30%) | 23 (70%) | 25 (69%) | 11 (31%) | 35 (51%) | 34 (49%) |
|  |  |  |  |  |  |  |
| Pregnancy type |  |  |  |  |  |  |
| Singleton | 45 (37%) | 78 (63%) | 70 (61%) | 44 (39%) | 115 (49%) | 122 (51%) |
| Twin | 2 (29%) | 5 (71%) | 4 (40%) | 6 (60%) | 6 (35%) | 11 (65%) |
|  |  |  |  |  |  |  |
| First pregnancy lasting 20 weeks or more |  |  |  |  |  |  |
| No | 27 (44%) | 34 (56%) | 35 (71%) | 14 (29%) | 62 (56%) | 48 (44%) |
| Yes | 20 (29%) | 49 (71%) | 39 (52%) | 36 (48%) | 59 (41%) | 85 (59%) |
|  |  |  |  |  |  |  |
| Mode of delivery |  |  |  |  |  |  |
| Caesarean | 28 (34%) | 54 (66%) | 39 (58%) | 28 (42%) | 67 (45%) | 82 (55%) |
| Vaginal | 19 (40%) | 29 (60%) | 35 (61%) | 22 (39%) | 54 (51%) | 51 (49%) |
|  |  |  |  |  |  |  |
| Breast feeding/expressing at their discharge |  |  |  |  |  |  |
| No | 2 (40%) | 3 (60%) | 5 (56%) | 4 (44%) | 7 (50%) | 7 (50%) |
| Yes | 41 (34%) | 80 (66%) | 66 (59%) | 45 (41%) | 107 (46%) | 125 (54%) |
| Baby not alive at mother’s discharge | 4 (100%) | - | 3 (75%) | 1 (25%) | 7 (88%) | 1 (13%) |
|  |  |  |  |  |  |  |
